# Comparison of calf muscle architecture between Asian children with spastic cerebral palsy and typically developing peers

**DOI:** 10.1371/journal.pone.0190642

**Published:** 2018-01-05

**Authors:** Ying Chen, Lu He, Kaishou Xu, Jinling Li, Buyun Guan, Hongmei Tang

**Affiliations:** 1 Department of Rehabilitation, Guangzhou Women and Children’s Medical Center, Guangzhou Medical University, Guangzhou, China; 2 Department of Ultrasonography, Guangzhou Women and Children’s Medical Center, Guangzhou Medical University, Guangzhou, China; Boston Children's Hospital / Harvard Medical School, UNITED STATES

## Abstract

**Objective:**

To compare the muscle thickness, fascicle length, and pennation angle of the gastrocnemius, soleus, and tibialis anterior between Asian children with spastic cerebral palsy (CP) and typically developing (TD) peers.

**Methods:**

This cross-sectional study involved a total of 72 children with hemiplegic CP (n = 24), and diplegic CP (n = 24) and their TD peers (n = 24). Muscle architecture was measured at rest using ultrasound. Clinical measures included gross motor function and a modified Ashworth scale.

**Results:**

The thicknesses of the tibialis anterior and medial gastrocnemius muscles were smaller in the affected calf of children with CP (*p*<0.05) than in those of their TD peers. Additionally, the lengths of the lateral gastrocnemius and soleus fascicle were shorter (*p*<0.05) in children with diplegic CP than in their TD peers. The fascicle length was shorter in the affected calf of children with CP (*p*<0.05) than in the calves of their TD peers or the unaffected calf of children with hemiplegic CP. However, the length of the lateral gastrocnemius fascicle was similar between the two legs of children with hemiplegic CP. The pennation angles of the medial gastrocnemius and soleus muscles were larger (*p*<0.05) in the affected calf in children with hemiplegic CP than in the calves of their TD peers. The fascicle length of the lateral gastrocnemius and the thickness of the soleus muscle were positively correlated with gross motor function scores in children with CP (*p*<0.05).

**Conclusions:**

Muscle thickness and fascicle length were lower in the affected tibialis anterior, gastrocnemius, and soleus in children with spastic CP. These changes may limit the ability to stand and walk, and indicate a need to strengthen the affected muscle.

## Introduction

The key feature of spastic cerebral palsy (CP) is spasticity, which causes significant alterations to muscle morphology and architecture over time despite the non-progressive nature of its brain lesions.[1,2] The spastic muscles are often shorter as a result of insufficient stretching. Studies have indicated that spasticity and decreased activity contribute to muscle weakness and imbalance, muscle atrophy resulting from disuse, muscle contracture, and a reduced range of motion in joints[3–7].

A muscle’s function is indicated by its architecture, including its muscle thickness, fascicle length, and pennation angle[8–12]. Studies have shown that muscle thickness can indirectly reflect muscle strength[13,14]. Moreover, motor impairment can induce changes in muscle architecture[5–7,9–18]. Muscle excursion is reflected in fascicle length, which impacts force generation and the maximum shortening speed[9]. Muscle morphology and structure are altered in children with CP to some degree as a result of secondary impairments, such as disuse, spasticity, and immobilization[15–23]. However, the data in this area of research remain limited. While some studies have suggested that the pennation angle of the medial gastrocnemius is significantly smaller in children with CP than in their typically developing (TD) peers[18,19], other studies have reported the opposite results[20–22]. The characteristics of its muscle architecture in children with CP therefore remains unclear[9,18–22]. However, muscle architecture is closely related to muscle excursion, the generation of force and power, and maximum muscle shortening speed. It is therefore very important to determine whether and how its muscle architecture is altered.

Ultrasound has recently become more widely used in musculoskeletal research because it is a relatively useful, quick, cheap and child-friendly assessment tool[18–26], even in light of its limitations (e.g., it provides only superficial data for the muscle region)[27] compared to diffusion tensor imaging and high-resolution anatomical MRI scans. A previous systematic review also provided good evidence backing the practical application of ultrasound as a valid measurement tool for determining the fascicle length and pennation angle[23]. Other reviews have also suggested that muscle thickness, length and volume tend to be smaller in children with CP[9,23]. However, these studies have provided very limited data related to muscle architecture in children with CP[9,18–23,28,29]. Furthermore, few reports have used ultrasound to explore the characteristics of the calf agonist (gastrocnemius and soleus) and antagonist (tibialis anterior) muscles in children with CP. The motor functions associated with these alterations in musculoskeletal development have not yet been fully explored.

Therefore, the objectives of this study were to use ultrasound to investigate the muscle thickness, fascicle length, and pennation angle of the calf agonist (the medial and lateral gastrocnemius and soleus) and antagonist (tibialis anterior) muscles between Asian children with spastic CP and their TD peers. Furthermore, we analyzed the correlations between muscle architecture and walking ability.

## Methods

### Participants

Children with hemiplegic and diplegic CP (age range, 2–13 y) who were treated in the Rehabilitation Department of Guangzhou Women and Children’s Medical Center from December 2014 to December 2015 were selected. Children with CP were excluded if they had been treated with serial casting, botulinum toxin injections or surgery to the legs during the previous 6 months. Twenty-four children with hemiplegic CP and 24 age-matched children with diplegic CP were recruited into the study among 97 eligible candidates. The participants with hemiplegic and diplegic CP were classified as level I (n = 29), II (n = 7) or Ⅲ (n = 12) according to the gross motor function classification system (GMFCS)[30] for dynamic equinus foot due to calf spasticity. Twenty-four age-matched TD peers were recruited from a general population of children with no known neurological or musculoskeletal problems. After consent forms were obtained from their parents, all participants were divided into the following three groups: a hemiplegic CP (HCP) group, a diplegic CP (DCP) group, and a group consisting of typically developing peers (TD). The study was approved by the medical ethical committee of Guangzhou Women and Children’s Medical Center.

### Design and experiment protocol

**Fig 1:**
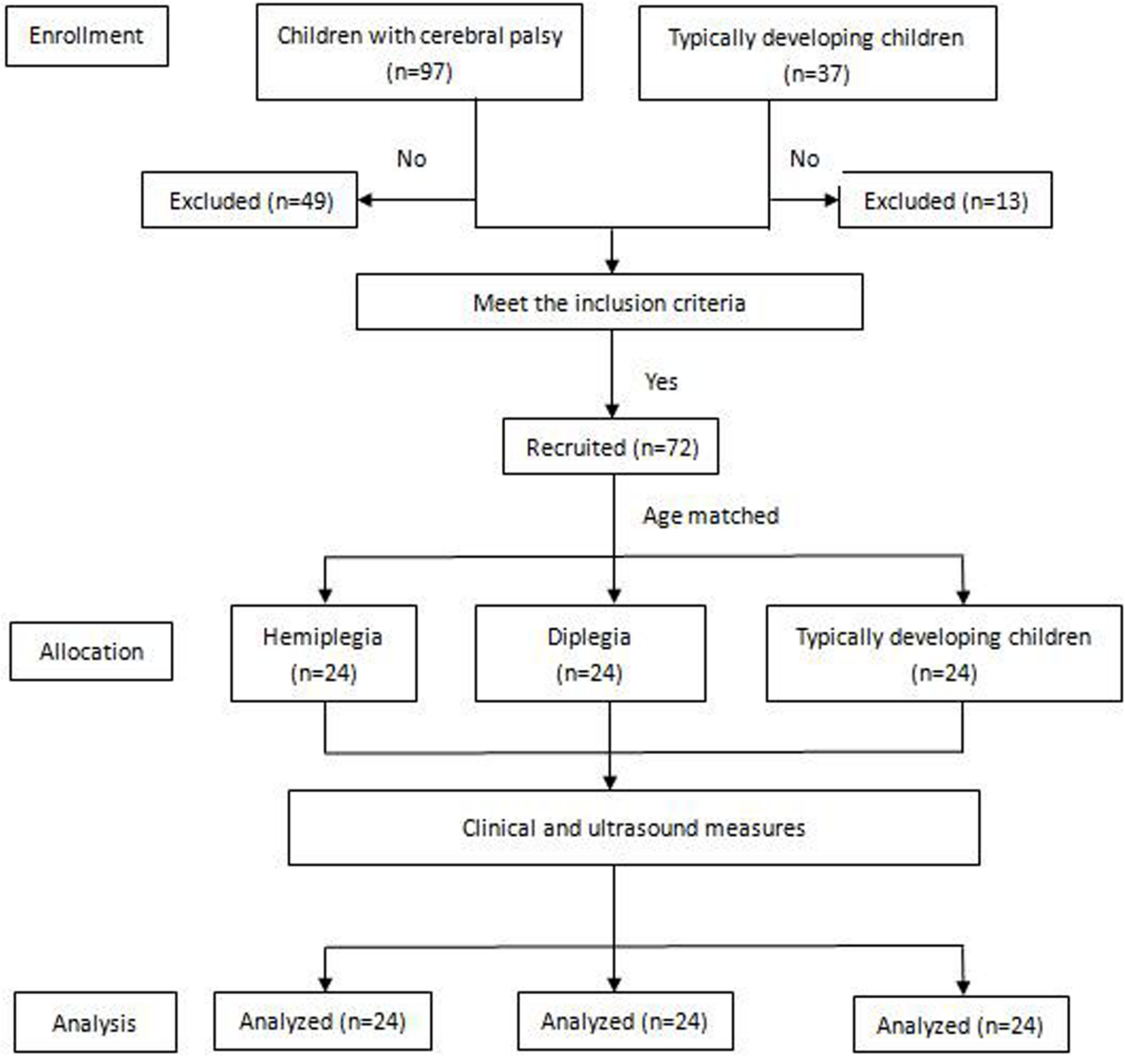
Flowchart of case screening and measurement protocols.

This was a cross-sectional study (Fig 1). The protocol and raw data for this trial is available as S1 Raw data and S1 Protocol. A between-group comparison and correlation analysis design was used. Anthropometric, clinical function and ultrasound measures were obtained during a single visit at our hospital.

Demographic characteristics were recorded for each participant and included age, gender, height, weight, body mass index, and calf length and circumference. Calf length was defined as the distance between the popliteal fossa and calcaneus. All children with hemiplegic and diplegic CP were assessed by two experienced physical therapists to determine the GMFCS, the D and E dimensions of the gross motor function measure (GMFM)[31], and the modified Ashworth scale (MAS)[32] for the ankle while the children maintained their knee in an extended position. GMFM is a standardized, observational assessment tool used to detect gross motor function in children with CP. The ability to stand and walk was tested in the D and E dimensions. MAS is the clinical spasticity scale that is most commonly used in cases of pediatric patients to assess muscle tone. It scores the resistance encountered in a specific muscle group when a limb is passively moved at one velocity through its range of motion. Ultrasound measurements of the calf were performed while the ankle of each child was in a resting position with the knee fully extended. Each participant lay prone on the examination couch with the distal portion of their legs off the plinth. The ankle angle was fixed by an assistant at an approximate plantarflexion angle of 20°. Both legs in the HCP and DCP groups and the right leg in the TD group were scanned according to the methods described in a previous study[33]. One sonographer with over 10 years of experience performed the ultrasound scans. One investigator analyzed and processed the data.

### Ultrasound measures

A computer-based ultrasound system equipped with an 11.0-MHz linear transducer with a 50-mm field of view (Siemens Acuson S2000^a^) was used during the measurement phases. Measurements were obtained from the calf muscle in the sagittal plane and horizontal planes. To avoid the effects of different system conditions, which could potentially alter the images, the performer standardized the setting of scanner and kept it constant during the examinations.

During ultrasound scanning of the tibialis anterior, the participants lay in bed and maintained a supine condition. The participants lay flat on the stomach during scanning of the medial and lateral gastrocnemius and soleus muscles. To obtain an objective outcome, the sonographer used a sufficient amount of contact gel for acoustic coupling and avoided exerting excessive pressure on the muscle. The probe was placed vertical to the surface of the largest circumference of the calf under examination. Two images, including a cross section and longitudinal section, were captured for each targeted muscle. Each parameter was measured 3 times in order to reduce error.

**Fig 2:**
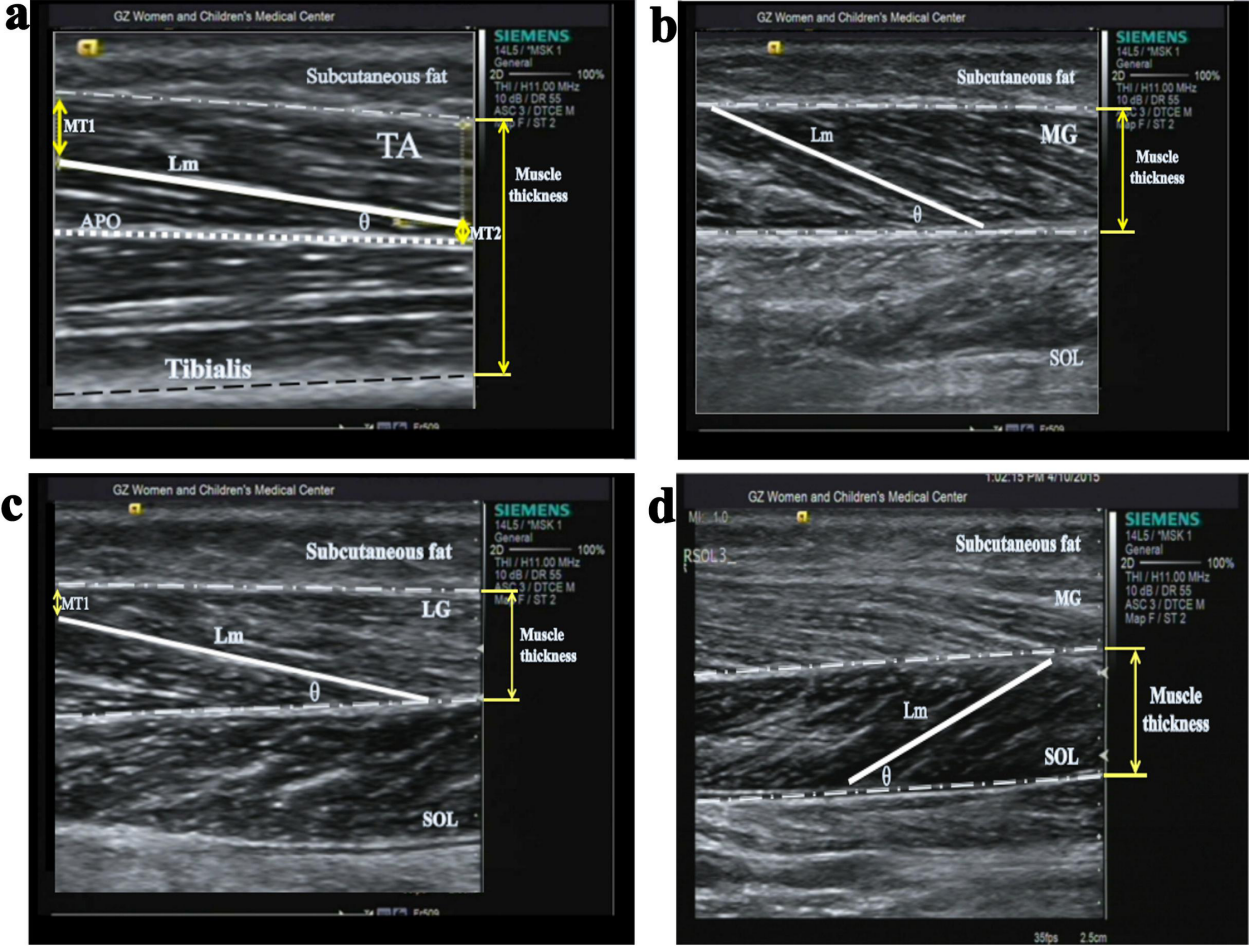
Ultrasonic images of calf muscles measured at rest: (a) tibialis anterior (TA), (b) medial gastrocnemius (MG), (c) lateral gastrocnemius (LG), and (d) soleus (SOL). θ indicates the pennation angle; APO, Aponeurosis; MT1 indicates the distance from the fiber distal end point to the superficial aponeurosis; MT2 indicates the distance from the fiber proximal end to the deep aponeurosis; Lm, Longest fiber length in the visible section by ultrasound.

The parameters that were analyzed included muscle thickness (muscle thickness = (muscle proximal thickness + muscle distal thickness)/2), fascicle length (fascicle length = longest visible fiber length + proximal muscle thickness/sinθ + distal muscle thickness/sinθ), and pennation angle. Measurements were obtained according to the recommendations of previous studies[9,23,33,34]. Muscle thickness was described as the straight-line distance between the superficial and deep aponeuroses. The longest visible fiber length observed on ultrasound was determined by measuring the straight-line distance between the superficial and deep aponeuroses that ran parallel to the lines of collagenous tissue that were visible on the image. Muscle proximal thickness was defined as the vertical distance between the proximal superficial and deep aponeuroses. Muscle distal thickness was defined as the perpendicular distance between the distal superficial and deep aponeuroses. The pennation angle (θ) was defined as the angle made by lines running along the fascicle length and the deep aponeurosis. Ultrasonic images of these calf measurements are shown in Fig 2.

### Statistical analysis

SPSS version 20.0 ^b^ was used to perform the analysis. If the values were normally distributed, the Independent-Samples t test, one-way ANOVA and a post hoc analysis (Bonferroni method if equal variances were assumed or Tamhane’s T2 method if equal variances were not assumed) were used. Otherwise, the Mann-Whitney U test, Kruskal-Wallis test and Chi-square test were used. The affected and unaffected limbs of the HCP group, the mean of both limbs in the DCP group, and the right limbs of the TD group were used in the analysis of muscle architecture data, calf length and circumference, and muscle spasticity. The Chi-square test was used to determine whether there were differences in gender and GMFCS levels. Correlations between muscle architecture and clinical functions were analyzed with Pearson’s (if a double normal distribution was assumed) or Spearman’s (if a double normal distribution was not assumed) correlation analysis. Significance was set at 0.05.

## Results

### Demographic, anthropometric and clinical function data

Demographic, anthropometric and clinical function data for the three groups are shown in Table 1. No significant differences (*p*>0.05) were found in age, gender, and body mass index among the three groups or in MAS scores for the ankle between the HCP and DCP groups. Height, weight, and calf circumference and length were significantly lower in the DCP group than in the TD group (*p*<0.01). The lengths of both calves and the circumference of the affected calf were significantly smaller in the HCP group than in the TD group, and calf circumference was smaller in the DCP group than in the HCP group (*p*<0.05). GMFCS levels and GMFM scores were significantly better in the HCP group than in the DCP group (*p*<0.05).

**Table 1:** Demographic, anthropometric and baseline data for the groups with hemiplegic cerebral palsy (HCP), diplegic cerebral palsy (DCP), and typically developing (TD). Results are shown as the mean (SD) or n (%).

Group	HCP (n = 24)	DCP (n = 24)	TD (n = 24)	*p*
Age (months)	56.3 (28.4)	54.4 (18.2)	57.9 (23.7)	0.613
Male	18 (75%)	12 (50%)	12 (50%)	0.128
Height (cm)	101.8 (17.5)	98.7 (13.9)	111.8 (12.6)	0.005
Weight (kg)	18.8 (10.8)	14.9 (4.0)	21.1 (6.3)	0.021
Body mass index	17.1 (2.6)	15.2 (2.1)	16.7 (3.4)	0.05
Calf length (cm)	24.5(5.1)^a^ /24.7 (5.1)^b^	23.0 (3.9)	27.2 (3.7)	0.004
Calf circumference (cm)	22.3(3.9)^a^ /22.8 (4.7)^b^	20.5 (2.1)	24.0 (2.4)	<0.001
Modified Ashworth scale	2.3 (0.1)	2.2 (0.1)	N/A	0.657
Gross motor function measure	73.0 (21.7)	39.5 (30.00)	N/A	<0.001
Gross motor function classification system level				
I	21 (88%)	8 (33%)	N/A	0.001
II	2 (8%)	5 (21%)		
III	1 (4%)	11 (46%)		

^a^On the affected side.

^b^On the unaffected side.

### Comparison of muscle architecture

**Fig 3:**
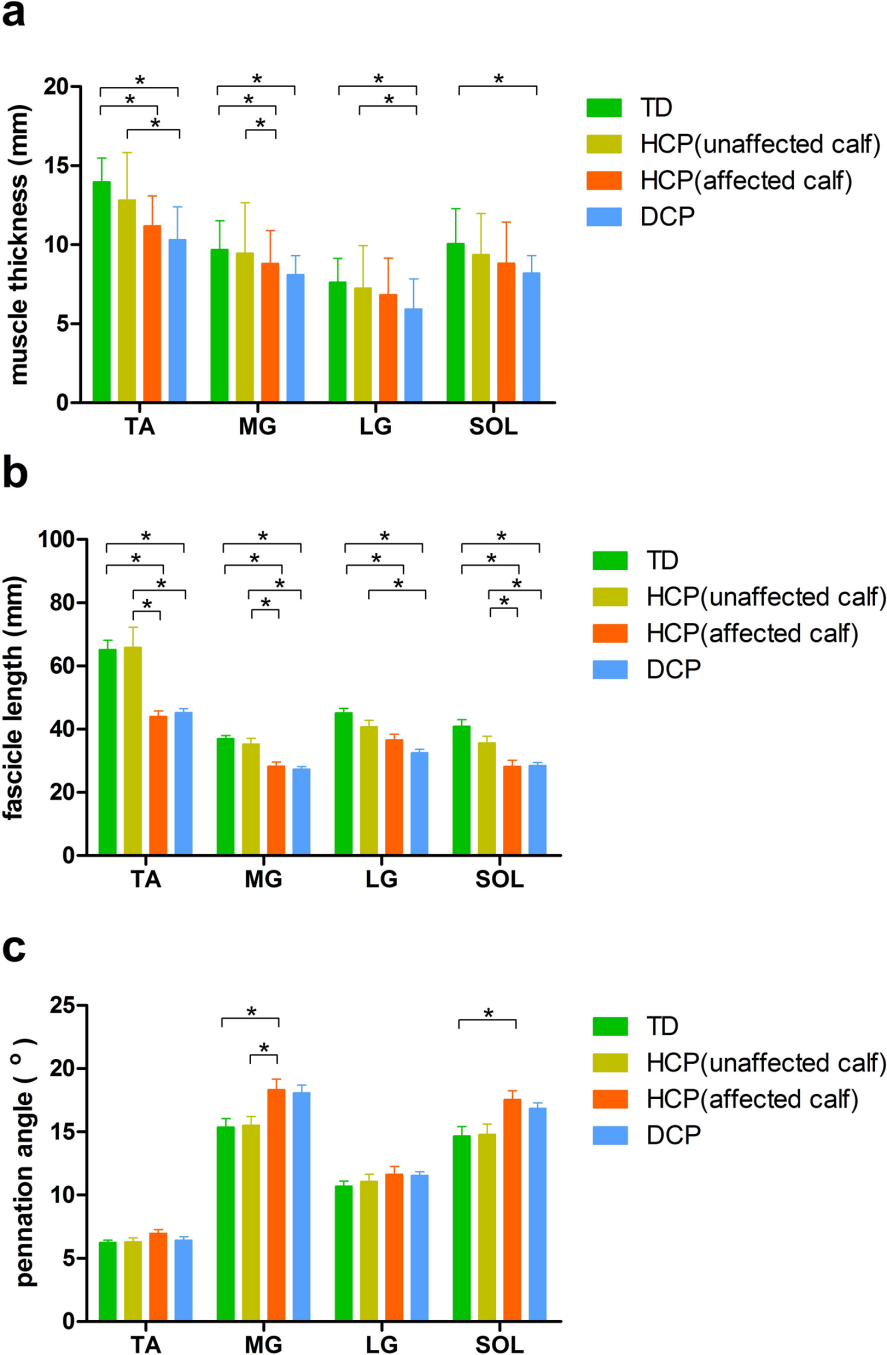
Comparison of at-rest muscle thickness (a), fascicle length (b), and pennation angle (c) in calf muscles among the groups with hemiplegic cerebral palsy (HCP), diplegic cerebral palsy (DCP), and typically developing (TD) (mean ± SD). TA, tibialis anterior; MG, medial gastrocnemius; LG, lateral gastrocnemius; SOL, soleus. *Significant group difference (*p*<0.05).

There were no significant differences (*p*>0.05, Table 2, Fig 3) in muscle thickness or the pennation angle of the tibialis anterior, lateral gastrocnemius, and soleus muscles or in the fascicle length of the lateral gastrocnemius muscle between the two legs of the HCP group. The fascicle length of the tibialis anterior, medial gastrocnemius, and soleus muscles and the thickness of the medial gastrocnemius muscle were significantly smaller on the affected side than on the unaffected side, and the pennation angle of the medial gastrocnemius muscle was smaller on the unaffected side than on the affected side (*p*<0.05, Table 2 and Fig 3).

**Table 2:** Data for muscle architecture parameters in the groups with hemiplegic cerebral palsy (HCP), diplegic cerebral palsy (DCP), and typically developing (TD). The results are shown as the mean (SD).

Group	HCP		DCP (n = 24)	TD (n = 24)	*p*
	Affected side (n = 24)	Unaffected side (n = 24)			
**Tibialis anterior**					
Muscle thickness (mm)	11.2 (1.9)	12.8 (3.0)	10.3 (2.1)	14.0(1.5)	<0.001
Pennation angle (°)	7.0 (1.4)	6.3 (1.5)	6.4 (1.9)	6.2 (1.0)	0.121
Fascicle length (mm)	43.9 (8.8)	65.9 (31.1)	45.2 (8.6)	65.1 (14.7)	<0.001
**Medial gastrocnemius**					
Muscle thickness (mm)	8.8 (2.1)	9.5 (3.2)	8.1 (1.2)	9.7 (1.8)	0.007
Pennation angle (°)	18.3 (4.0)	15.5 (3.3)	18.1 (4.3)	15.4 (3.3)	0.011
Fascicle length (mm)	28.3 (6.3)	35.3 (8.7)	27.3 (5.9)	37.0 (4.8)	<0.001
**Lateral gastrocnemius**					
Muscle thickness (mm)	6.9 (2.3)	7.3 (2.7)	5.9 (1.9)	7.6 (1.5)	<0.001
Pennation angle (°)	11.6 (3.0)	11.1 (2.8)	11.6 (2.1)	10.7 (2.0)	0.470
Fascicle length (mm)	36.6 (8.8)	40.7 (10.3)	32.5 (7.6)	45.1 (7.2)	<0.001
**Soleus**					
Muscle thickness (mm)	8.8 (2.6)	9.4(2.6)	8.2 (1.1)	10.1 (2.2)	0.005
Pennation angle (°)	17.6 (3.4)	14.8(3.9)	16.8 (3.0)	14.7 (3.6)	0.010
Fascicle length (mm)	28.2 (9.6)	35.6(10.1)	28.5 (6.6)	40.9 (10.4)	<0.001

No significant differences were found in any of the muscle architecture parameters between the targeted muscles in the TD group and the targeted muscles on the unaffected side of the HCP group. There was also no difference in the thickness of the lateral gastrocnemius and soleus muscles or in the pennation angle of the tibialis anterior and lateral gastrocnemius muscles between the TD group and the affected side of the HCP group (*p*>0.05, Table 2, Fig 3). The thickness and fascicle length of the tibialis anterior and medial gastrocnemius muscles were significantly smaller in the affected side of the HCP group than in the TD group, and the fascicle length of the lateral gastrocnemius and soleus muscles were significantly smaller on the affected side of the HCP group than in the TD group. However, the pennation angles of the medial gastrocnemius and soleus muscles were significantly larger on the affected side of the HCP group than in the TD group (*p*<0.05, Table 2, Fig 3).

The thickness of the tibialis anterior and lateral gastrocnemius muscles and the fascicle length of all of the targeted muscles were significantly smaller in the DCP group than on the unaffected side of the HCP group (*p*<0.05, Table 2, Fig 3).

No significant differences were found in the pennation angle of the calf muscles between the DCP and TD groups (*p*>0.05, Table 2, Fig 3). However, the thickness and fascicle length of the targeted muscles were significantly smaller in the DCP group than in the TD group (*p*<0.05, Table 2, Fig 3).

### Correlation analysis

MAS scores were not well correlated with any of the muscle architecture parameters in children with CP (*p*>0.05). However, lateral gastrocnemius fascicle length and soleus muscle thickness were correlated with GMFM scores in all children with CP (r = 0.414 and r = 0.402, respectively; *p*<0.01).

## Discussion

This is the first investigation to compare the muscle architecture of the calf agonist (gastrocnemius and soleus) and antagonist (tibialis anterior) muscles between Asian children with CP and their typical peers. In this study, we demonstrated that muscle thickness and fascicle length were significantly lower in the affected than in the TD muscles and in the affected than in the unaffected muscles for the tibialis anterior, gastrocnemius, and soleus muscles. However, the pennation angles of the medial gastrocnemius and soleus muscles were significantly higher on the affected side of children with hemiplegic CP than in their TD peers. In addition, our results also indicate that the ability to stand and walk is associated with lateral gastrocnemius fascicle length and soleus muscle thickness.

Skeletal muscles are subject to reshaping in the body and can be adapted by a variety of positive and negative stimulations, such as strength training, disuse, spasticity, and immobilization[15–17]. Data in both animal and human models[15,16] have shown that muscle size and fascicle length become smaller in response to disuse and immobilization. This effect on muscle thickness and fascicle length may contribute to muscle weakness and imbalance[8,11,35,36]. An increasing number of studies have reported that muscle thickness is smaller and fascicle length is shorter in the gastrocnemius muscle in children with spastic CP. Noble et al[29,37] suggested that a reduction in muscle size during childhood could contribute to the deterioration of mobility observed in teenagers and adults with spastic CP. The similar findings reported in the present study suggest that the decrease observed in muscle thickness and fascicle length in the calf of CP patients might be the result of spasticity, disuse and limited activity. The timing of onset of secondary impairments remains unclear in children with CP. A previous study suggested that muscle changes could occur at an earlier age than is generally believed[28]. Thus, it is very important for children with CP to perform early targeted activities to optimize these adaptive changes in muscle architecture and function.

Muscle thickness is positively correlated with force capacity[28,35–37]. Previous studies have indicated that the gastrocnemius muscle could be affected by spasticity and disuse[19,20,22]. We further found that both muscle thickness and fascicle length were smaller in the tibialis anterior and soleus and that these changes were correlated with a poor ability to stand and walk. These changes might limit gross motor function in children with CP. The findings presented in this study are consistent with those described in previous reports[19–22,38]. The results of this study show that the thickness of the affected calf is associated with lower mobility and even atrophy. These effects may result in muscle fibrosis, which can decrease muscle strength and contraction elasticity[39]. Decreased muscle strength has also been observed in children with CP, suggesting limited activation[11]. This lack of activation and plasticity could compromise the muscle[40]. Specific activation patterns may induce substantial fiber type plasticity and result in smaller muscle thickness in children with CP[4,41,42]. Studies have suggested that targeted strength training should be implemented in these children to reverse the progress of muscle disuse and atrophy, to support the development of the corticospinal system, and to increase muscle recruitment[43–45].

Based on the clinical experience, motor dysfunctions may be influenced more by limitations related to mechanical factors in soft tissues than to neurological factors in central command disorders as children with CP have grown up to be youths and adults. Fascicle length is especially important when analyzing muscle architecture because it is highly related to secondary musculoskeletal contracture development, which can severely limit the initial length of muscle contraction and excursion[9,28,40,46,47]. In this study, we show that the fascicle length in the affected calf was shorter than that observed in both TD peers and the unaffected side of children with hemiplegic CP. This alteration could contribute to the tip-toe gait, decreased range of motion, and decreased gait speed observed in CP. The results of previous reports regarding the changes that occur in fascicle length in children with spastic CP have been inconsistent[18–21]. Because fascicle length changes as muscles contract, studies have yielded conflicting results. Another potentially influential factor may be the fact that fascicle length is dependent on joint position. Mohagheghi et al[20] suggested that reported fascicle lengths were shorter when they were not normalized to bone length. In our study, the ankle joint was maintained in a consistent position during ultrasound scanning of the targeted muscles. In addition, the targeted muscle was also kept relaxed during the testing procedure. Normalizing fascicle length therefore did not seem to be an important factor in this study. The results of the present study (Fig 3B) also showed that the gastrocnemius and soleus muscles exhibited relatively shorter fascicle lengths, whereas the tibialis anterior muscle had a comparatively longer fascicle length. This finding is consistent with a previous study[48] in which it was suggested that ankle plantar flexors have a relatively short fascicle length that is ideal for high muscle force, whereas ankle dorsiflexors have comparatively longer fascicle lengths and are therefore well-suited for high muscle excursion.

Muscle fibers often do not run the entire muscle length from origin to insertion parallel to the action of the muscle, but are instead at the pennation angle. Although Malaiya et al[19] and Shortland et al[18] found that the pennation angles of the medial gastrocnemius muscle were significantly smaller in spastic CP than in TD peers, this was not evident while the ankle joint was in a resting position. Moreover, some previous studies[22,49] have reported finding non-significant differences in pennation angle among muscles on the affected and unaffected sides of CP patients and both sides of healthy participants. Generally, a large pennation angle indicates that more fibers are packed into a muscle, allowing it to produce more force, whereas a small pennation angle might be positively related to an increase in the velocity of muscle shortening during contraction. However, some fascicular shortening is produced by fascicles rotating about their insertion points in the aponeuroses, which can be caused by brain lesion-induced hypertonicity, and this process increases the pennation angle. In the present study, we show that the pennation angles of the affected medial gastrocnemius and soleus muscles were significantly larger in children with hemiplegic CP than in their TD peers, suggesting that the velocity of shortening in the affected muscle is limited in children with hemiplegic CP. Additionally, an increased pennation angle might neutralize the negative influence of atrophy on the affected muscle. However, the functional impact of the observed difference in pennation angle remains unclear and is worth further exploration.

Muscle thickness and fascicle length were decreased in the affected tibialis anterior, gastrocnemius, and soleus muscles. Furthermore, GMFCS levels and GMFM scores were poorer in the DCP group, indicating that a smaller muscle thickness and a shorter fascicle length decrease muscle strength, resulting in the loss of good functions in the legs.

With regard for the effects of CP, the calf muscles have been the most well-studied muscle group because they are easily induced to contract in children with CP. Most studies have used ultrasound to measure muscle architecture parameters. While ultrasound imaging is a specialized and complicated process, MRI is used less often because it is expensive and problematic to perform in that it requires prolonged relaxation, which is difficult to achieve in a primarily pediatric population. A previous systematic review suggested that ultrasound imaging is accurate when measuring the architectural features of human skeletal muscles in vivo if certain conditions are met, including the relaxation of and maintenance of a static position in joints[23] In the present, all ultrasounds were performed by a sonographer with over 10 years of muscle-scanning experience who exhibited good reliability and validity.

### Study limitations

First, we investigated the muscle architectures of the calf only while the ankle was in a resting position with the knee extended. Defining the muscle architecture associated with different ankle joint angles during voluntary contraction will require further investigation. Second, we did not measure tendon length. Some previous studies[50,51] have suggested that the ratio of muscle fascicle length to tendon length might be another parameter required to evaluate muscle function. Because this ratio is not fixed for a specific muscle but instead changes during normal muscle growth from childhood to adulthood [52], it might be altered in children with CP. Third, while fascicle length was not normalized to calf length in this study, we suggest that this issue remains controversial[20]. Finally, the differences in height, weight, calf length and circumference among the groups may have contributed to bias in this study. It is possible that the inclusion of a larger number of participants would provide greater power to the presented results.

## Conclusions

Muscle thickness and fascicle length are decreased in the affected tibialis anterior, gastrocnemius, and soleus muscles of children with CP. These changes may limit the ability of these individuals to stand and walk. Additionally, these alterations indicate that there is a need to strengthen the affected muscles in children with spastic CP. Further studies are needed to determine whether muscle growth and architecture can be improved in children with spastic CP by interventions such as treatment with botulinum toxin and strength training.

## Supporting information

S1 Raw data (XLS)

S1 Protocol (DOC)

## Abbreviations

CP: cerebral palsy
DCP: diplegic cerebral palsy
GMFCS: gross motor function classification system
GMFM: gross motor function measure
HCP: hemiplegic cerebral palsy
MAS: modified Ashworth scale
MRI: magnetic resonance imaging
TD: typically developing
