# Supplementary material for: Comparison of calf muscle architecture between Asian children with spastic cerebral palsy and typically developing peers
Source: PLoS One. 2018 Jan 5;13(1):e0190642. doi: 10.1371/journal.pone.0190642 (PMC5755874; doi:10.1371/journal.pone.0190642)
Supplement: S1 Protocol — (DOC) [file pone.0190642.s002.doc]

**Comparison of calf muscle architecture between Asian children with spastic cerebral palsy and typically developing peers**

**Summary**

**Background**

The key feature of spastic cerebral palsy (CP) is spasticity, which causes significant alterations to muscle morphology and architecture over time despite the non-progressive nature of its brain lesions. The spastic muscles are often shorter as a result of insufficient stretching. Studies have indicated that spasticity and decreased activity contribute to muscle weakness and imbalance, muscle atrophy resulting from disuse, muscle contracture, and a reduced range of motion in joints. A muscle’s function is indicated by its architecture, including its muscle thickness, fascicle length, and pennation angle. Studies have shown that muscle thickness can indirectly reflect muscle strength. Moreover, motor impairment can induce changes in muscle architecture. Muscle excursion is reflected in fascicle length, which impacts force generation and the maximum shortening speed. Muscle morphology and structure are altered in children with CP to some degree as a result of secondary impairments, such as disuse, spasticity, and immobilization. However, the data in this area of research remain limited. While some studies have suggested that the pennation angle of the medial gastrocnemius is significantly smaller in children with CP than in their typically developing (TD) peers, other studies have reported the opposite results. The characteristics of its muscle architecture in children with CP therefore remains unclear. However, muscle architecture is closely related to muscle excursion, the generation of force and power, and maximum muscle shortening speed. It is therefore very important to determine whether and how its muscle architecture is altered.

**Purpose**

To use ultrasound to investigate the muscle thickness, fascicle length, and pennation angle of the calf agonist (the medial and lateral gastrocnemius and soleus) and antagonist (tibialis anterior) muscles between Asian children with spastic CP and their TD peers. Furthermore, we analyzed the correlations between muscle architecture and walking ability.

**Study end-points**

**Primary outcome measures:**

Ultrasound measurements of the calf were performed while the ankle of each child was in a resting position with the knee fully extended. Each participant lay prone on the examination couch with the distal portion of their legs off the plinth. The ankle angle was fixed by an assistant at an approximate plantarflexion angle of 20°. Both legs in the HCP and DCP groups and the right leg in the TD group were scanned according to the methods described in a previous study. Ultrasound measures included muscle thickness (muscle thickness = (muscle proximal thickness + muscle distal thickness)/2), fascicle length (fascicle length = longest visible fiber length + proximal muscle thickness/sinθ + distal muscle thickness/sinθ), and pennation angle.

Demographic characteristics were recorded for each participant and included age, gender, height, weight, body mass index, and calf length and circumference. Calf length was defined as the distance between the popliteal fossa and calcaneus.

All children with hemiplegic and diplegic CP were assessed by two experienced physical therapists to determine the GMFCS, the D and E dimensions of the gross motor function measure (GMFM), and the modified Ashworth scale (MAS) for the ankle while the children maintained their knee in an extended position.

**Study design**

This cross-sectional study involved a total of 72 children with hemiplegic CP (n=24), and diplegic CP (n=24) and their TD peers (n=24). Muscle architecture was measured at rest using ultrasound. Clinical measures included gross motor function and a modified Ashworth scale. After consent forms were obtained from their parents, all participants were divided into the following three groups: a hemiplegic CP (HCP) group, a diplegic CP (DCP) group, and a group consisting of typically developing peers (TD). The study was approved by the medical ethical committee of Guangzhou Women and Children’s Medical Center.

**Study population**

Seventy-two patients.

**Inclusion criteria**

Children with hemiplegic and diplegic CP (age range, 2-13 y) who were treated in the Rehabilitation Department of Guangzhou Women and Children’s Medical Center from December 2014 to December 2015 were selected. Twenty-four children with hemiplegic CP and 24 age-matched children with diplegic CP were recruited into the study among 97 eligible candidates.

Twenty-four age-matched TD peers were recruited from a general population of children with no known neurological or musculoskeletal problems.

**Exclusion criteria**

Children with CP were excluded if they had been treated with serial casting, botulinum toxin injections or surgery to the legs during the previous 6 months.

**Interventions**

Not applicable

**Risks and inconveniences**

Not applicable

**Statistical analyses: sample size and power calculation**

A total of 62 patients has to be recruited. Considering a drop-out rate of 15%, a total of 72 children with hemiplegic CP (n=24), and diplegic CP (n=24) and their TD peers (n=24).

**Study flowchart**


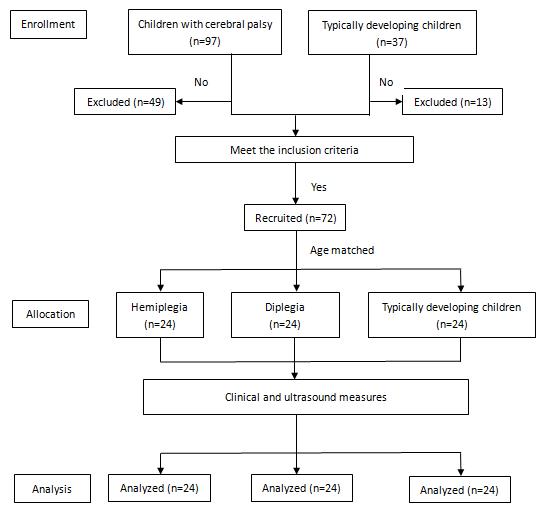


**Signature page**

**Investigators**

**Ying Chen^1^**† **MD, Lu He^1^**† **MD, Kaishou Xu^1*^ PhD, Jinling Li^1^ MD, Buyun Guan^2^ MD, Hongmei Tang^1^ PhD**

1 Department of Rehabilitation, Guangzhou Women and Children’s Medical Center, Guangzhou Medical University, Guangzhou, 510120, China

2 Department of Ultrasonography, Guangzhou Women and Children’s Medical Center, Guangzhou Medical University, Guangzhou, 510120, China

**† These authors contributed equally to this work**

**Ying Chen,**

Staff member, Department of Rehabilitation,

Guangzhou Women and Children’s Medical Center, Guangzhou Medical University.

**Lu He**,

Staff member, Department of Rehabilitation,

Guangzhou Women and Children’s Medical Center, Guangzhou Medical University.

**Kaishou Xu**,

Director, Department of Rehabilitation,

Guangzhou Women and Children’s Medical Center, Guangzhou Medical University.

**Jinling Li**

Staff member, Department of Rehabilitation,

Guangzhou Women and Children’s Medical Center, Guangzhou Medical University.

**Buyun Guan**

Director, Department of Ultrasonography,

Guangzhou Women and Children’s Medical Center, Guangzhou Medical University

**Hongmei Tang**

Staff member, Department of Rehabilitation,

Guangzhou Women and Children’s Medical Center, Guangzhou Medical University

**Guangzhou 14.09.2013**
